# Supplementary material for: Sociodemographic and Clinical Profile of Long COVID-19 Patients, and Its Correlation with Medical Leave: A Comprehensive Descriptive and Multicenter Study
Source: Healthcare (Basel). 2023 Sep 27;11(19):2632. doi: 10.3390/healthcare11192632 (PMC10572288; doi:10.3390/healthcare11192632)
Supplement: Supplementary file 1 [file healthcare-11-02632-s001.zip › healthcare-2598161-supplementary.pdf]

Estudio EPICOVID-AP21

jegonla@gmail.com Cambiar de cuenta

No compartido

* Indica que la pregunta es obligatoria

Consentimiento informado

A continuación, indique si está de acuerdo con las siguientes indicaciones para participar en el estudio:

Comprendo que mi participación es voluntaria y puedo retirarme del estudio cuando quiera y si lo solicito se eliminará de la base de datos la información suministrada sin que ello tenga ninguna repercusión en mi persona. *

- Estoy de acuerdo
- No estoy de acuerdo

Presto libremente mi conformidad para participar en el estudio *

- ◯ Si
- ◯ No (gracias por su atención, cierre la página del cuestionario)

1. ¿Cuál fue la prueba diagnóstica o test con la que le detectaron la infección por SARS-CoV-2? *

- ☐ PCR
- ☐ Antígeno rápido
- ☐ Test serológico
- ☐ Ninguna de ellas, fue por sospecha clínica

2. Si le hicieron test de seguimiento de la enfermedad ¿Cuál fue la prueba? *

- ☐ PCR
- ☐ Antígeno rápido
- ☐ Test serológico
- ☐ No me hicieron prueba de seguimiento

3. Si le hicieron test de seguimiento de la enfermedad ¿Cuál fue el resultado?

- ◯ Positivo
- ◯ Negativo
- ◯ Inconcluyente
- ◯ No me hicieron prueba de seguimiento

4. ¿Cuanto tiempo ha durado su proceso hasta la recuperación total del COVID? *

- ◯ De 56 a 79 días
- ◯ De 80 a 99 días
- ◯ De 100 a 119 días
- ◯ De 120 a 159 días
- ◯ De 160 a 200 días
- ◯ De 201 a 250 días
- ◯ Mas de 250 días
- ◯ Aún no me he recuperado del todo del COVID persistente

5. ¿Quién le comunicó a usted la primera vez que padecía COVID persistente?

- ☐ Médico de familia
- ☐ Neumólogo
- ☐ Medico internista
- ☐ Dermatólogo
- ☐ Neurólogo

6. Indique los síntomas que ha presentado desde que se infectó por la COVID19 *

	Si	No
Dolor de garganta	☐	☐
Dolor de cabeza, cefalea	☐	☐
Dolor de articulaciones	☐	☐
Dolor muscular	☐	☐
Cansancio no habitual, fatiga	☐	☐
Dificultad para respirar o sensación de ahogo	☐	☐
Falta o perdida de olfato	☐	☐
Falta o perdida del gusto	☐	☐
Tos	☐	☐
Sensación de falta de aire, disnea	☐	☐
Fiebre	☐	☐
Sudoración	☐	☐
Escalofríos, tiritera	☐	☐
Congestión nasal	☐	☐
Afonía, ronquera	☐	☐

Malestar general	☐	☐
Dolor torácico	☐	☐
Dolor espalda	☐	☐
Sensación de opresión en el pecho	☐	☐
Diarrea	☐	☐
Dolor estomacal	☐	☐
Dolor abdominal	☐	☐
Vómitos	☐	☐
Náuseas	☐	☐
Perdida de apetito	☐	☐
Pérdida de peso	☐	☐
Hipotermia (temperatura corporal baja)	☐	☐
Molestias oculares(conjuntivitis, ojo seco, visión borrosa, cuerpo extraño, congestión)	☐	☐
Eritema facial (lesión rojiza en la cara)	☐	☐
Pseudocongelación en extremidades (acrosíndrome, lesiones parecidas a los sabañones)	☐	☐
Esputos o flemas (secresión bronquial)	☐	☐

Hemoptisis (esputos con sangre)	☐	☐
Hinchazón, inflamación de los dedos	☐	☐
Picores (prurito)	☐	☐
Ronchas o eccemas en la piel (erupciones, urticaria o habones)	☐	☐
Temblores	☐	☐
Mareos	☐	☐
Vertigos	☐	☐
Convulsiones	☐	☐
Pérdida de memoria	☐	☐
Confusión mental	☐	☐
Falta de concentración/déficit de atención	☐	☐
Niebla mental	☐	☐
Dificultad para dormir	☐	☐
Estréss postraumatico	☐	☐
Parestesias	☐	☐
Dificultad para tragar	☐	☐
Pitidos de oído, acúfenos		

Ojos secos	☐	☐
Conjuntivitis	☐	☐
Palpitaciones	☐	☐
Presión arterial alta	☐	☐
Presión arterial baja	☐	☐
Pérdida del cabello	☐	☐
Disfunción eréctil (hombres)	☐	☐
Trastornos menstruales (mujeres)	☐	☐

Trastornos menstruales (mujeres) ☐ ☐

7. Indique los síntomas que persisten en la actualidad desde que se infectó por la COVID19. Si se encuentra en la actualidad ya asintomático, no responda a estas pregunta y pase a la pregunta 9

	Si	No
Dolor de garganta	☐	☐
Dolor de cabeza, cefalea	☐	☐
Dolor de articulaciones	☐	☐
Dolor muscular	☐	☐
Cansancio no habitual, fatiga	☐	☐
Dificultad para respirar o sensación de ahogo	☐	☐
Falta o perdida de olfato	☐	☐
Falta o perdida del gusto	☐	☐
Tos	☐	☐
Sensación de falta de aire, disnea	☐	☐
Fiebre	☐	☐
Sudoración	☐	☐
Escalofríos, tiritera	☐	☐
Congestión nasal	☐	☐

Afonía, ronquera	☐	☐
Malestar general	☐	☐
Dolor torácico	☐	☐
Dolor espalda	☐	☐
Sensación de opresión en el pecho	☐	☐
Diarrea	☐	☐
Dolor estomacal	☐	☐
Dolor abdominal	☐	☐
Vómitos	☐	☐
Náuseas	☐	☐
Perdida de apetito	☐	☐
Pérdida de peso	☐	☐
Hipotermia (temperatura corporal baja)	☐	☐
Molestias oculares(conjuntivitis, ojo seco, visión borrosa, cuerpo extraño, congestión)	☐	☐
Eritema facial (lesión rojiza en la cara)	☐	☐
Pseudocongelación en extremidades (acrosíndrome, lesiones parecidas a los sabañones)	☐	☐

[illegible]

Esputos o flemas (secresión bronquial)	☐	☐
Hemoptisis (esputos con sangre)	☐	☐
Hinchazón, inflamación de los dedos	☐	☐
Picores (prurito)	☐	☐
Ronchas o eccemas en la piel (erupciones, urticaria o habones)	☐	☐
Temblores	☐	☐
Mareos	☐	☐
Vertigos	☐	☐
Convulsiones	☐	☐
Pérdida de memoria	☐	☐
Confusión mental	☐	☐
Dificultad para dormir	☐	☐
Falta de concentración/déficit de atención	☐	☐
Niebla mental	☐	☐
Estréss postraumatico	☐	☐
Parestesias	☐	☐
Dificultad para tragar	☐	☐

Pitidos de oído, acúfenos	☐	☐
Ojos secos	☐	☐
Conjuntivitis	☐	☐
Palpitaciones	☐	☐
Presión arterial elevada	☐	☐
Presión arterial baja	☐	☐
Pérdida del cabello	☐	☐
Disfunción eréctil (hombres)	☐	☐
Trastornos menstruales (mujeres)	☐	☐

8. Indique cuales son los síntomas que le está/n resultando o le han resultado más incapacitante/s para realizar sus actividades de la vida diaria.

	Si	No
Dolor de garganta	☐	☐
Dolor de cabeza, cefalea	☐	☐
Dolor de articulaciones	☐	☐
Dolor muscular	☐	☐
Cansancio no habitual, fatiga	☐	☐
Dificultad para respirar o sensación de ahogo	☐	☐
Falta o perdida de olfato	☐	☐
Falta o perdida del gusto	☐	☐
Tos	☐	☐
Sensación de falta de aire, disnea	☐	☐
Fiebre	☐	☐
Sudoración	☐	☐
Escalofríos, tiritera	☐	☐
Congestión nasal	☐	☐
Afonía, ronquera	☐	☐

Malestar general	☐	☐
Dolor torácico	☐	☐
Dolor espalda	☐	☐
Sensación de opresión en el pecho	☐	☐
Diarrea	☐	☐
Dolor estomacal	☐	☐
Dolor abdominal	☐	☐
Vómitos	☐	☐
Náuseas	☐	☐
Perdida de apetito	☐	☐
Pérdida de peso	☐	☐
Hipotermia (temperatura corporal baja)	☐	☐
Molestias oculares(conjuntivitis, ojo seco, visión borrosa, cuerpo extraño, congestión)	☐	☐
Eritema facial (lesión rojiza en la cara)	☐	☐
Pseudocongelación en extremidades (acrosíndrome, lesiones parecidas a los sabañones)	☐	☐
Esputos o flemas (secreción bronquial)		

bronquial)

Hemoptisis (esputos con sangre)	☐	☐
Hinchazón, inflamación de los dedos	☐	☐
Picores (prurito)	☐	☐
Ronchas o eccemas en la piel (erupciones, urticaria o habones)	☐	☐
Temblores	☐	☐
Mareos	☐	☐
Vertigos	☐	☐
Convulsiones	☐	☐
Pérdida de memoria	☐	☐
Confusión mental	☐	☐
Falta de concentración/déficit de atención	☐	☐
Niebla mental	☐	☐
Dificultad para dormir	☐	☐
Estréss postraumatico	☐	☐
Parestesias	☐	☐
Dificultad para tragar	☐	☐
	☐	☐

Pitidos de oído, acúfenos	☐	☐
Ojos secos	☐	☐
Conjuntivitis	☐	☐
Palpitaciones	☐	☐
Presión arterial arterial alta	☐	☐
Presión arterial baja	☐	☐
Pérdida del cabello	☐	☐
Pérdida del cabello	☐	☐

9. ¿Cuál fue el primer síntoma o signo que presentó al comienzo del cuadro clínico de la COVID19? *

- Dolor de garganta
- Dolor de cabeza cefalea
- Dolor de articulaciones
- Dolor muscular
- Cansancio no habitual, fatiga
- Dificultad para respirar o sensación de ahogo
- Falta o perdida de olfato
- Falta o perdida del gusto
- Tos
- Diarrea
- Vómitos
- Náuseas
- Dolor estomacal
- Dolor abdominal
- Perdida de apetito
- Fiebre
- Sudoración
- Escalofríos, tiritera
- Hipotermia (temperatura corporal baja)
- Afonía, ronquera
- Congestión nasal (mucosidad)
- Esputos (secreción bronquial)

- Hemoptisis (esputos con sangre)
- Malestar general
- Dolor torácico
- Dolor espalda
- Sensación de opresión en el pecho
- Molestias oculares(conjuntivitis, ojo seco, visión borrosa, cuerpo extraño, congestión)
- Eritema facial (enrojecimiento en la cara)
- Pseudocongelación en extremidades (acrosíndrome, como sabañones)
- Ronchas o eccemas en la piel (erupciones, urticaria o habones)
- Picores (prurito)
- Mareos
- Vertigos
- Temblores
- Convulsiones
- Pérdida de memoria
- Confusión mental
- Falta de concentración/déficit de atención
- Otro:

10. ¿Cuáles son los órganos, sistemas o áreas que se han visto afectadas en su proceso de COVID19? (admite mas de una opción) *

	Si	No
Afectación del estado general	○	○
Sistema nervioso	○	○
Esfera mental (psicológico/emocional)	○	○
Sistema respiratorio	○	○
Aparato digestivo	○	○
Sistema endocrino	○	○
Aparato locomotor	○	○
Sistema cardiovascular	○	○
Ocular	○	○
ORL	○	○
Dermatológico	○	○
Nefrourológico	○	○
Hematológico (coagulación)	○	○
Ninguno	○	○

11. ¿Cómo considera que es su estado de salud general actualmente? (desde 0, el peor estado de salud, hasta 10, el mejor estado de salud) *

- 0 ○
- 1 ○
- 2 ○
- 3 ○
- 4 ○
- 5 ○
- 6 ○
- 7 ○
- 8 ○
- 9 ○
- 10 ○

12. En comparación con su estado de salud antes de padecer la COVID19, ¿En que grado ha cambiado su estado de salud tras sufrir COVID persistente? (poner 0 si sigue igual y hasta 10, el máximo empeoramiento que podía usted imaginar) *

- 0 ○
- 1 ○
- 2 ○
- 3 ○
- 4 ○
- 5 ○
- 6 ○
- 7 ○
- 8 ○
- 9 ○
- 10 ○

13. En una escala de 0 al 10, ¿Cuál diría que es su estado de incapacidad actual tras padecer la COVID19 persistente? (marcar 0 si no siente ningún grado de incapacidad hasta 10, la máxima incapacidad que cabe imaginar).

- 0 ○
- 1 ○
- 2 ○
- 3 ○
- 4 ○
- 5 ○
- 6 ○
- 7 ○
- 8 ○
- 9 ○
- 10 ○

14. ¿En qué grado diría que le ha afectado a su calidad de vida la COVID19 persistente? (marcar 0 si no le afecta nada hasta 10, la máxima afectación que cabe imaginar). Si no presenta o ha presentado COVID persistente no responda esta pregunta

- 0 ○
- 1 ○
- 2 ○
- 3 ○
- 4 ○
- 5 ○
- 6 ○
- 7 ○
- 8 ○
- 9 ○
- 10 ○

15. ¿Ha presentado usted neumonía tras el diagnostico de la COVID19? *

- ○ Si
- ○ No

16. ¿Ha estado usted ingresado en un hospital a consecuencia de la COVID19? *

- ○ Si
- ○ No

17. ¿Estuvo ingresado en la UCI?

- ○ Si
- ○ No

18. Indique si padece actualmente alguna de estas enfermedades *

	Si	No
Hipertensión arterial	○	○
Diabetes Mellitus	○	○
EPOC (enfermedad pulmonar obstructiva crónica)	○	○
Asma	○	○
Hiperlipemia (colesterol alto)	○	○
Sobrepeso u obesidad	○	○
Inmunodepresión (defensas bajas, VIH,..)	○	○
Cáncer	○	○
Insuficiencia respiratoria	○	○
Insuficiencia renal	○	○
Insuficiencia cardiaca	○	○
Insuficiencia hepática	○	○
Depresión	○	○
Ansiedad	○	○
Enfermedad mental (psicóticas, neurosis,		

Alhzeimer,..)		
Enfermedad autoinmune (colitis ulcerosa,..)	○	○
Enfermedad vascular (ACVA, ictus, arteriopatía)	○	○
Cardiopatía (fibrilación auricular, valvulopatía, IAM, angor, HVI,..)	○	○
Enfermedad endocrina (hipotiroidismo,...)	○	○
Enfermedad neurológica	○	○
Enfermedad neurológica	○	○

19. ¿Fuma usted? (exfumador: que hubiera fumado durante al menos un año y llevara más de un año sin fumar) *

- No, nunca he fumado
- No, pero soy ex-fumador
- Si, esporadicamente
- Si, todos los días

20. ¿Consume usted alcohol? *

- Si
- Alguna vez
- No

21. ¿Le han puesto la vacuna para la COVID19? *

- ◯ Si
- ◯ No (pase a la pregunta 26)

22. ¿Cuál fue la vacuna o vacunas que le pusieron? *

- ☐ Ninguna
- ☐ Pfizer
- ☐ AstraZeneca
- ☐ Moderna
- ☐ Jansen
- ☐ Otro:

Si se ha vacunado, ¿Cuántas dosis de la vacuna le han puesto?

- ◯ Una
- ◯ Dos
- ◯ Tres
- ◯ Cuatro

23. ¿Ha presentado algún efecto adverso a la vacuna de los que se indican a continuación? (si no se ha vacunado, no responda a esta pregunta)

	Si	No
Fiebre	○	○
Quebrantamiento general	○	○
Dolor en la zona de la inyección	○	○
Cansancio/fatiga	○	○
Dolor de espalda	○	○
Dolor muscular	○	○
Dolor articular	○	○
Pérdida de apetito	○	○
Náuseas	○	○
Vómitos	○	○
Dolor estomacal	○	○
Dolor abdominal	○	○
Trastornos de coagulación	○	○
Parálisis facial	○	○
Pérdida del gusto	○	○

Pérdida del olfato	○	○
Hipertensión arterial	○	○
Accidente vascular cerebral	○	○
Dermatitis	○	○
Otros	○	○
Dolor de cabeza	○	○
Mareos	○	○
Escalofrios	○	○
Picor	○	○
Trombosis	○	○
Trastornos de la regla/menstruación (mujeres)	○	○

24. ¿Ha notado algún cambio en su cuadro clínico tras haberse vacunado de la COVID19?

- No me he vacunado
- Ha empeorado
- Sigo igual que antes
- He notado mejoría
- Ha desaparecido la sintomatología

25. Indique el tratamiento que está siguiendo usted actualmente para la COVID19 persistente

	Si	No
Analgésicos	○	○
Antiinflamatorios	○	○
Ansiolíticos	○	○
Antidepresivos	○	○
Anticoagulantes	○	○
Corticoides	○	○
Calcifediol (vitamina D)	○	○
Otros	○	○
Ninguno	○	○

26. Señale su grupo sanguíneo *

- A
- B
- AB
- O

27. Señale su factor RH *

- Positivo
- Negativo

Atrás Siguiente Borrar formulario

Nunca envíes contraseñas a través de Formularios de Google.

Este contenido no ha sido creado ni aprobado por Google. Notificar uso inadecuado - Términos del Servicio - Política de Privacidad

Google Formularios
